# Encoding Information into Polyethylene Glycol Using an Alcohol-Isocyanate “Click” Reaction

**DOI:** 10.3390/ijms21041318

**Published:** 2020-02-15

**Authors:** Lajos Nagy, Ákos Kuki, Tibor Nagy, Bence Vadkerti, Zoltán Erdélyi, Levente Kárpáti, Miklós Zsuga, Sándor Kéki

**Affiliations:** 1 Department of Applied Chemistry, Faculty of Science and Technology, University of Debrecen, H-4032 Debrecen, Hungary; nagy.lajos@science.unideb.hu (L.N.); akos@science.unideb.hu (Á.K.); nagy.tibor@science.unideb.hu (T.N.); vadkerti.bence@science.unideb.hu (B.V.); zsuga.miklos@science.unideb.hu (M.Z.); 2 Doctoral School of Chemistry, University of Debrecen, H-4032 Debrecen, Hungary; 3 Department of Solid State Physics, Faculty of Science and Technology, University of Debrecen, H-4002 Debrecen, Hungary; zoltan.erdelyi@science.unideb.hu; 4 Department of Organic Chemistry, Faculty of Pharmacy, Semmelweis University, H-1092 Budapest, Hungary; karpati.levente@pharma.semmelweis-univ.hu

**Keywords:** monomethoxypolyethylene glycol (mPEG), aliphatic isocyanates, encoding and decoding binary information, MALDI-TOF MS

## Abstract

In this article, the capability of encoding information using a homologous series of monodisperse monomethoxypolyethylene glycols (mPEG), with a number of ethylene oxide units ranging from n_EO_ = 5 to 8, and monodisperse linear aliphatic isocyanates containing a number of CH_2_ units from 3 to 7, is demonstrated. The “click” reaction of the two corresponding homologous series yielded 20 different isocyanate end-capped polyethylene glycol derivatives (mPEG-OCONHR) whose sodiated adduct ion’s nominal *m*/*z* values spanned from 360 to 548, providing an average ca. 8 *m*/*z* unit for the storage of one-bit information. These mPEG-OCONHR oligomers were then used to encode information in binary sequences using a 384-well MALDI sample plate and employing the common dried-droplet sample preparation method capable of encoding 20 bit, i.e., 2.5 byte information in one spot, was employed. The information stored in the spots was read by MALDI-TOF MS using the *m*/*z* value of the corresponding mPEG-OCONHR oligomers. The capability of the method to store data was demonstrated by writing and reading a text file, visualizing a small picture and capturing a short audio file written in Musical Instrument Digital Interface (MIDI) sequence. Due to the very large similarities in the chemical structures of the encoding oligomers and their “easy to be ionized” property, as well as their very similar ionization efficiencies, the MALDI-TOF MS signal intensities from each compound was so strong and unambiguous that complete decoding could be performed in each case. In addition, the set of the proposed encoding oligomers can be further extended to attain higher bit “densities”.

## 1. Introduction

The need for encoding and decoding information for safety, communication or any other reasons such as storage of data is almost as old as humankind itself. For example, ancient data storage included various notched sticks, carved bones, scribed tablets and painted rocks, among others. Undoubtedly, one of the oldest and simplest information storage techniques is the painting of pictures on the walls of caves and rocks [1] but a more complex data storage “device” called “quipu” (an assembly of colored knotted cotton cords) was developed and used by the ancient Inca [2]. Recently, however, in the era of digital storage, there is an increasing need for alternatives to existing data storage technologies, not primarily due to them exceeding their current speeds and the space required to store a given amount of data but rather to eliminate some of their weaknesses associated with, e.g., energy consumption required for storage and long-term stability [3].

A very promising alternative way to the current digital storage system is the use of natural [4] and synthetic (macro)molecules [5]. Undoubtedly, DNA is the highest capacity natural macromolecule so far capable of storing information as high as 455 exabytes/gram but encoding information in DNA, especially in the case of long strands, is a very slow, complex and expensive process [6,7,8,9]. Another approach, similar to encoding in DNA, is based on synthetic copolymers in which information is encoded into the sequence of the comonomers [10,11,12,13,14,15] and the information is read by mass spectrometric methods, although other strategies for encoding molecules and reading have also been reported [16,17]. Moreover, the synthesis of long copolymer chains to store a bigger data set may run into difficulties. In addition, reading out data from the encoded copolymers requires MS instruments equipped with MS/MS facility, otherwise the information cannot be read [18,19,20]. Very recently, a low molecular weight synthetic metabolome mixture [21] and a carefully designed series of oligopeptides [22] were used to demonstrate encoding of texts and pictures. In this strategy, the presence or absence of a given compound (a given *m*/*z*) encoded one bit of information. Although this approach proved to be very efficient, there may be some weaknesses in using compounds of different structures for encoding and decoding. (i) designing the attainable *m*/*z* range, the mass difference between the adjacent peaks, and the "*m*/*z*" density of the encoded information are not straightforward tasks. (ii) Since reading is performed by mass spectrometric methods, the ionization efficiency and, thus the signal intensity markedly depend on the structure of the encoding compound. This issue makes the automatic decision on the status "present" or "not present" ambiguous, introducing bit errors during the reading process. (iii) adduct ions with overlapping *m*/*z* values may be formed and/or charge competition reactions can take place under mass spectrometric conditions between the members of encoding compounds, hence, hindering the *m*/*z* values of some encoding compounds from appearing in the corresponding mass spectra.

In this article, we offer another strategy for encoding, which eliminates the cumbersome details above. According to this approach, to obtain encoded oligomers we use monodisperse monomethoxypolyethylene glycol (mPEG) of varying lengths reacted with monodisperse alkylisocyanate with different numbers of CH_2_ units. Thus, the resulting oligomers containing urethane bonds are structurally very similar to each other and their *m*/*z* values reveal the combination of 44 and 14 mass units. The capability of this approach to store data was demonstrated by writing and reading a text file, visualizing a small picture and capturing a short audio file written in Musical Instrument Digital Interface (MIDI) sequence.

## 2. Results and Discussion

### 2.1. Theoretical Requirement for Encoding Oligomers Composed of Two Different Homologous Series

If the presence or absence of given *m*/*z* values encodes information, it is essential to determine the accessible *m*/*z* range, the average *m*/*z* spacing that encodes a given amount of information (i.e., one bit) and, whether there are overlapping *m*/*z* values that reduce the number of encoding peaks in the corresponding *m*/*z* range.

Focusing on the family of compounds that are combinations of two homologous series, e.g., copolymers or the present mPEG-OCONHR oligomers, the accessible *m*/*z* range can easily be given by Equations (1) and (2).

(*m*/*z*)_low_ = n_min_M_1_ + m_min_M_2_ + M_end_ + M_cat_ (1)

(*m*/*z*)_high_ = n_max_M_1_ + m_max_M_2_ + M_end_ + M_cat_ (2)

where (*m*/*z*)_low_ and (*m*/*z*)_high_ are the lower and the upper *m*/*z* value accessible, respectively, M_1_ and M_2_ are the masses of repeat units, n_min_ and m_min_ stand for the lowest number of repeat unit 1 and 2, respectively, while n_max_ and m_max_ represent the highest number of repeat unit 1 and 2, respectively, and M_1_ ≠ M_2_. M_end_ and M_cat_ are the masses of the end-group and the cation attached to the chain.

The number of encoding compounds (N_total_) formed from two homologous series can be represented by Equation (3).

N_total_ = (n_max_ − n_min_ + 1)(m_max_ − m_min_ + 1) − χ (3)

where χ is the number of overlapping *m*/*z* values.

Overlapping occurs when the nominal *m*/*z* value is the same for two n and m combinations [23]. Although it is possible, due to the different elemental compositions, to separate these peaks by means of a mass spectrometer with high resolving power, however, it is more appropriate to avoid such potential bit-errors by leaving either of the two combinations out from encoding. Overlapping of the nominal *m*/*z* values occurs when Equation (4) is satisfied.

Δn/Δm = M_2_′/M_1_′ (4)

where M_1_′ and M_2_′ are the reduced masses of the repeat units obtained after simplification.

In the case of our encoding mPEG-OCONHR oligomer M_1_′ and M_2_′ are 44 and 14, respectively and thus after simplification Equation (4) reduces to Equation (5).

Δn/Δm = 7/22(5)

Hence, Equation (5) predicts that a pair of polymer chains with their number of ethylene oxide (EO) and methylene groups differing by 7 EO and 22 CH_2_ repeat units have the same nominal *m*/*z* value. For example, if n_max_−n_min_ = 14 and m_max_−m_min_ = 29, then according to Equation (5), 64 pairs of mPEG-OCONHR oligomers (i.e., χ = 64) with equal nominal *m*/*z* values are formed. Thus, the total number of mPEG-OCONHR oligomers that can be used for encoding based on Equation (3) is 386. Moreover, if we take into account that all of the available monodisperse mPEGs range from mPEG_3_ to mPEG_12_ and alkylisocyanate covers a number of CH_2_ units from 2 to 18, the total number of encoding mPEG-OCONHR oligomers is 170 (and χ = 0, since Equation (5) is not fulfilled). It can also be surmised that the other advantage of using monodisperse mPEG and alkylisocyanate is that a relatively high number encoding oligomers can be produced without overlapping *m*/*z* values.

**Figure 1:**
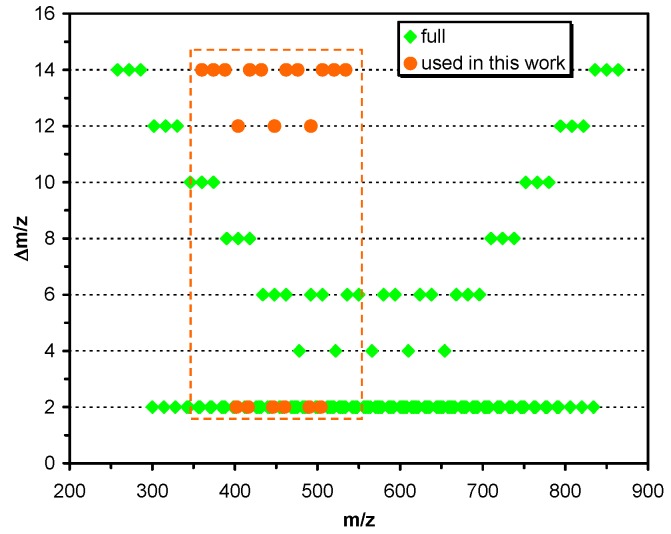
The variation of the *m*/*z* differences in the series composed of different numbers of ethylene oxide (EO) and CH_2_ units as a function of their *m*/*z* values. “Full” indicates the complete set of masses of oligomers that can be produced using all of the available monodisperse mPEG and alkylisocyanates with different chain lengths, while the marked region indicates those that were used in this study.

It is demonstrated in Figure 1 how the Δ*m*/*z* values (the mass difference between the adjacent peaks) of this oligomer series (light green) and that of the encoding oligomers used in this work (light brown) are distributed over the *m*/*z* range. As seen in Figure 1, indeed, there are no overlapping *m*/*z* values and the Δ*m*/*z* values are spanning from 2 to 14. In our study, the monodisperse mPEG used range from mPEG_5_ to mPEG_8_ and alkylisocyanate cover a number of CH_2_ units from 3 to 7, providing twenty encoding oligomers mass spaced by 14, 12 and 2 units. Hence, applying all the 20 polymers 20 bit, i.e., 2.5 byte information can be encoded in one spot. Furthermore, the nominal *m*/*z* values of the sodiated adduct ions of these mPEG-OCONHR oligomers cover a range from *m*/*z* 360 to 548, providing an average ca. 8 *m*/*z* unit for the storage of one-bit information.

### 2.2. The Relative Ion Intensities of mPEG-OCONHR Oligomers

**Figure 2:**
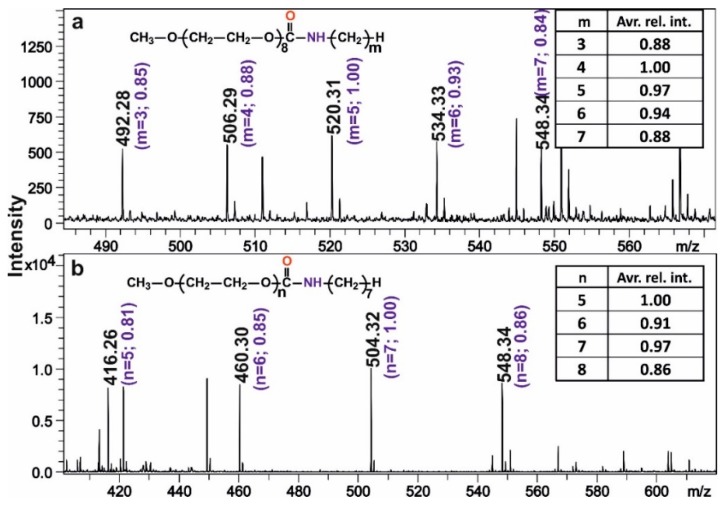
MALDI-TOF MS spectra and intensities of mPEG-OCONR oligomers with mPEG_8_ and alkylisocyanates (m = 3 to 7) (**a**) and alkylisocyanate with m = 7 and mPEG_n_ (*n* = 5 to 8) (**b**). Numbers in the brackets indicate the relative intensities for the case of (**a**,**b**) while the inset tables show the average relative intensities by taking into account the other members of the series.

In order to decode the data with high recovery, i.e., with minimal or no bit errors, it is important that the encoding compounds, in addition to their “easy to be ionized” nature, are to be similar ionization probabilities and no charge competition reaction can occur under mass spectrometric conditions. To evaluate these tasks, monodisperse mPEGs were reacted with the equimolar mixture of alkylisocyanates with m = 3–7. Similarly, monodisperse alkylisocyanates were also separately reacted with the mixture consisting of mPEG_n_ in equimolar ratio from *n* = 5–8. The MALDI-TOF MS spectra obtained on these mixtures together with the relative and the average relative intensities are depicted in Figure 2. As seen in Figure 2, indeed, due to the very high similarity of the chemical structure of the encoding oligomers, they appear closely equal MALDI-TOF MS intensities. As it turns out from the inset tables of Figure 2, the average relative intensities span from 0.86 to 1 indicating that ionization efficiencies do not depend on the chain lengths and that no charge competition reactions are taking place under MALDI conditions.

### 2.3. Storing Information in mPEG-OCONHR Oligomers

**Figure 3:**
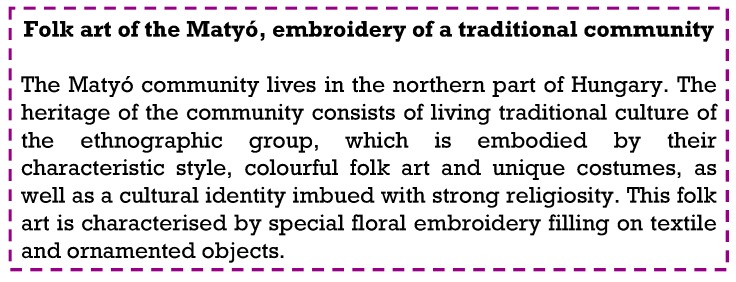
The text of “Matyó” embroidery encoded by means of mPEG-OCONHR polymers and decoded by MALDI-TOF MS.

To demonstrate the encoding capability of mPEG-OCONHR oligomers, first, a text shown in Figure 3 was encoded and decoded.

The text shown in Figure 3 was decoded with 100% efficiency, i.e., no bit errors occurred during reading owing to the high signal to noise ratio.

**Figure 4:**
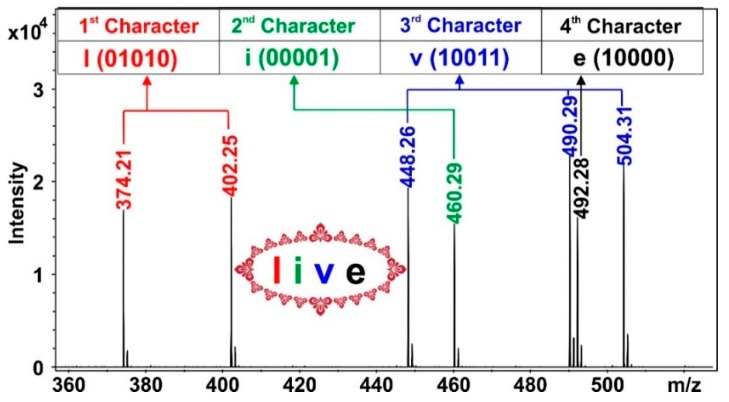
Decoding (reading) the “live” word from the text on the “Matyó” embroidery (shown in Figure 3) by MALDI-TOF MS.

For encoding, all of the 20 mPEG-OCONHR oligomers were employed. To encode characters, the variation in the length of the alkylisocyanate chains was utilized. Accordingly, 5 bits (since there are five different length alkylisocyanate) represent a character. Hence, 32 different characters could be encoded using the available alkylisocyanates. These 32 characters were letters of the English alphabet (26), the Hungarian letter “ó”, space, dot, comma, an indicator of capital letters (this indicates that a capital letter follows) and the enter. Furthermore, the variation in the mPEG lengths was utilized to indicate the order of the characters encoded in one spot, i.e., in one mass spectrum. The increasing mPEG chain lengths represented directly the order of characters, i.e., the first, the second, the third and the fourth character as going from mPEG_5_, to mPEG_8_, respectively. Note that in our study four mPEG chain lengths were available, thus, altogether four characters (20 bits) could be stored in one spot. As an example, decoding the “live” word from the text shown in Figure 3 by MALDI-TOF MS is presented in Figure 4.

According to Figure 4, the presence of two peaks at *m*/*z* 374 (*n* = 5, *m* = 4) and 402 (*n* = 5, *m* = 6) and absence of those at *m*/*z* 360 (*n* = 5, *m* = 3), 388 (*n* = 5, *m* = 5) and 416 (*n* = 5, *m* = 7) decode the letter “l” (01010). In addition, peaks at *m*/*z* 374 and 402 also reveal that mPEG_5_ (*n* = 5) is present indicating that letter “l” is the first character among the four characters encoded in the spot. Similarly, e.g., the presence of a peak at *m*/*z* 492 (*n* = 8, *m* = 3) and absence of the other four *m*/*z* values (506, 520, 534, 548; *n* = 8, *m* = 4–7) determine letter “e” (10000) and it is also unambiguous that this letter is the fourth (since mPEG_8_ is present). The text presented in Figure 3 contains 476 characters evenly distributed among 119 spots carrying 297.5 bytes of information altogether. The codes used for encoding the text are presented in the Supplementary Materials.

**Figure 5:**
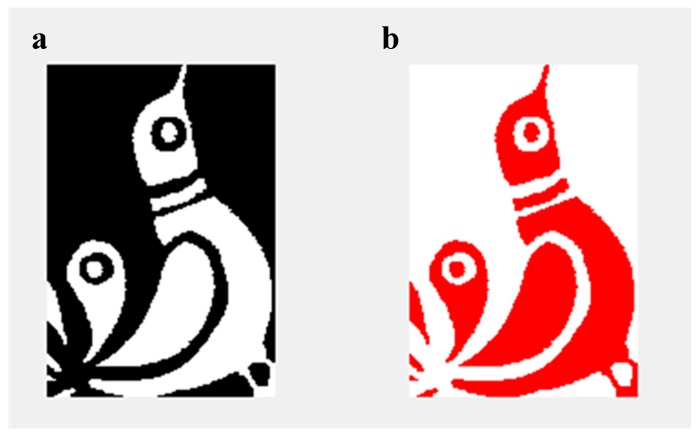
An original picture on a “Matyó” embroidery (bird pattern, 126×184 pixel) used for encoding information (**a**) and its Run Length Encoding (RLE) compressed version as decoded by MALDI-TOF MS (**b**).

The mPEG-OCONHR oligomers were also applied for binary, encoding a picture of 3006 bytes consisting of 126x184 pixels (Figure 5a).

To reduce the size of the picture shown in Figure 5a, we used the so-called Run Length Encoding (RLE) algorithm [24]. Accordingly, the first one bit in a byte represents the color of the pixel (colored or white), and the following 7 bits determine how many pixels of this color are in a row. Using this algorithm, the size of the picture was reduced to 952 bytes covering 381 spots in the sample plate. The reading of information was also achieved with 100% efficiency and the decoded picture is shown in Figure 5b.

The mPEG-OCONHR oligomers were also employed to encode a Musical Instrument Digital Interface (MIDI) file containing the tune of an old popular American ballad. In this demonstration, the spots encode the bits of the MIDI binary file in sequence, which were also recovered with 100% efficiency. The recovered MIDI file has been uploaded as Supplementary Materials.

It should be noted, however, that bit errors may occur in cases when encodings are performed manually (e.g., accidental inappropriate mixing of the encoding solutions due to human factors, the errors were lower than 0.3% in all cases). In contrast, by means of using robotics for encoding, this drawback can be eliminated or highly reduced on one hand, and the encoding is performed much faster, on the other hand. It is also of great importance that the spots had remained stable under ambient circumstances: after more than one month the readings were achieved with 100% recoveries and no noticeable decrease in the absolute intensities was observed. The time available for the investigation of stability was relatively short, yet, based on the chemical bonds involved in these encoding oligomers, it can be surmised that the spots will most likely preserve the information stored in them for a long time in the absence of conditions such as light, moisture, oxidative or reductive circumstances, etc.

Furthermore, it is worth estimating and comparing the theoretical limit of data storage density to those of classic information tools. Thus, taking into consideration the size of the laser beam (ca. 20 μm), and that the irradiated area is approximately 400 μm^2^ (containing 20 bits), the calculated bit density is 5 Mbit/cm^2^, which is higher than that of a conventional floppy disc (3.5 inch, 1.44 MB, 0.36 Mbit/cm^2^), but significantly lower than that of a contemporary hard-disk drive (207,700 Mbit/cm^2^).

## 3. Experimental Section

### 3.1. Chemicals

Propyl isocyanate (99%), butyl isocyanate (98%), pentyl isocyanate (97%), hexyl isocyanate (97%) and heptyl isocyanate (98%) were obtained from Sigma Aldrich (Darmstadt, Germany) and used as received. Monodisperse monomethoxypolyethylene glycols with a number of ethylene oxide units of 5, 6, 7 and 8 were purchased form JENKEM Technology (Beijing, China) and dried at 60 °C in vacuum overnight. 2,5-dihydroxybenzoic acid and methanol were obtained from Sigma Aldrich (Darmstadt, Germany) and used as received.

### 3.2. Preparation of Encoding Oligomers

**Figure 6:**
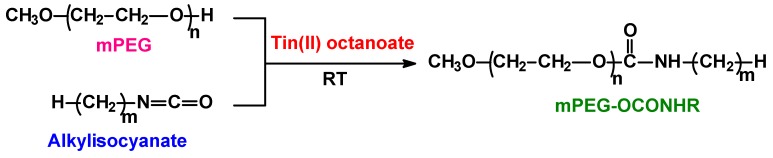
Reaction scheme for the urethane forming reaction of monomethoxypolyethylene glycols (mPEGs) with alkylisocyanates.

The corresponding monomethoxypolyethylene glycol (100 mg) (mPEG) was dissolved in 300 μL toluene containing tin(II) 2-ethyl-hexanoate at a concentration of 10 mg/mL. To this solution a calculated amount of alkylisocyanate was added in one-fold stoichiometric excess to mPEG and allowed to react for 5 hours, then the solution was further diluted 100-fold with methanol (to quench the unreacted alkylisocyanate). The reaction scheme for the urethane forming reaction of mPEG with alkylisocyanate is shown in Figure 6.

### 3.3. Matrix-Assisted Laser Desorption/Ionization Time-of-Flight Mass Spectrometry (MALDI-TOF MS)

The MALDI-TOF MS measurements, i.e., the reading of the information, were carried out with a Bruker Autoflex Speed mass spectrometer (Bruker Daltonik, Bremen, Germany) operating in the reflectron mode. For all of the measurements 19 kV (ion source voltage 1), 16.65 kV (ion source voltage 2), 21 kV (reflector voltage 1) and 9.55 kV (reflector voltage 2) voltages were used. The solid phase laser (355 nm) was operated at 500 Hz with 60% laser attenuation and 2000 shots were summed. The spectra were externally calibrated using polyethylene glycol (M_n_ = 400 g/mol). The information spots were prepared with 2,5-dihydroxybenzoic acid (DHB, 20 mg/mL) and sodium trifluoroacetate (NaTFA, 5 mg/mL) dissolved in a mixture of methanol and distilled water (80/20 *V*/*V*) using 50 μL matrix/NaTFA solution mixed with a series of encoding mPEG-OCONHR oligomer solutions of 5 μL each. From these solutions aliquots of 0.25 μL were deposited onto the stainless steel 384-well sample plate and allowed to air-dry.

To encode a picture, a text and Musical Instrument Digital Interface (MIDI) sequence (song) of 381, 119 and 151 spots were used in the MALDI sample plate. Mixing and deposition of the solutions to the sample plate were performed manually in each case.

### 3.4. Reading Information by MALDI-TOF MS

To read the information stored in the spots, Visual Basic script for automatic data processing was written. This script searches in the MS data file for all of the 20 *m*/*z* values used for encoding. If the particular *m*/*z* value is found, it is designated by “1”, or if not, by “0” bit and all bites are saved into an output file for further processing. The criteria for assigning a peak with one bit is that its intensity should at least be 25% of the maximum intensity of the peak that appeared in the corresponding MS spectrum and the *m*/*z* values should be within ±0.1 mass unit error. The information stored in the output files was decoded by a home-made program using “The professional Free Pascal RAD IDE” software in the environment of the Lazarus system. The Visual Basic script for automatic data processing is presented in the Supplementary Materials.

## 4. Conclusions

In this study, oligomers obtained by the reaction of monodisperse monomethoxypolyethylene glycol and alkylisocyanate with varying chain lengths were used to encode information in the spots of a MALDI sample plate. It was demonstrated that due to the high similarity of the chemical structure of the mPEG-OCONHR oligomers, the oligomer chains showed equal ionization efficiencies and no charge competition reactions occurred under MALDI conditions. Furthermore, the MS peaks of the encoding oligomers appeared with high intensity and thus, reading was achieved by MALDI-TOF-MS with high accuracy and efficiency. The data storage capability of the synthesized twenty mPEG-OCONHR oligomers was demonstrated for writing and reading a picture, a text and a MIDI sequence. These findings highlight the fact that the combination of two homologous series offers a unique way to obtain monodisperse oligomers with high chemical structure similarities capable of encoding information and designed mass range and mass spacing. In addition, the present set of mPEG-OCONHR oligomers can also be extended with higher molecular weight homologous to attain higher storage capacity and density. It is to be noted that at the present state such encoding and decoding approaches cannot compete with the existing digital storage technologies. Moreover, considering its unique nature e.g., no energy consumption for data storage and potential long-term stability under normal circumstances, it still can be a valuable alternative information storage strategy in addition to the current data storage systems.

## Supplementary Materials

Supplementary materials can be found at https://www.mdpi.com/1422-0067/21/4/1318/s1.
