# Supplementary material for: Encoding Information into Polyethylene Glycol Using an Alcohol-Isocyanate “Click” Reaction
Source: Int J Mol Sci. 2020 Feb 15;21(4):1318. doi: 10.3390/ijms21041318 (PMC7072859; doi:10.3390/ijms21041318)
Supplement: Supplementary file 1 [file ijms-21-01318-s001.zip › ijms-713728-supplementary-2.7/Supplementary Materials.pdf]

Supplementary Materials

Encoding information into polyethylene glycol using alcohol-isocyanate „click" reaction

Lajos Nagy[1], Ákos Kuki[1], Tibor Nagy[1], Bence Vadkerti[1,2], Zoltán Erdélyi[3], Levente Kárpáti[4], Miklós Zsuga[1], Sándor Kéki[1*]

[1]Department of Applied Chemistry, Faculty of Science and Technology, University of Debrecen, H-4032 Debrecen, Egyetem tér 1., Hungary

[2]University of Debrecen, Doctoral School of Chemistry, H-4032 Debrecen, Egyetem tér 1., Hungary

[3]Department of Solid State Physics, Faculty of Science and Technology, University of Debrecen, H-4002 Debrecen, Bem tér 8/a, Hungary

[4]Department of Organic Chemistry, Faculty of Pharmacy, Semmelweis University H-1092 Budapest, Hőgyes Endre u. 7.

Table of Contents

FlexAnalysis Visual Basic script for automatic data processing..2

Codes for encoding the text..3

FlexAnalysis Visual Basic script for automatic data processing

```
'#Uses "d:\methods\flexAnalysisMacroModules\FATools.obm"
Option Explicit
Option Base 1
Private aAnaSpec() As TAnaSpec
Private nSpectra%

Sub Main
nSpectra = Tools.GetSelectedAnaSpec(aAnaSpec)
If nSpectra = 0 Then
Err.Raise 1,,"No spectrum selected"
End If

Dim i%
Dim rp As Variant
rp=Array(360.199,374.215,388.231,402.247,404.225,416.263,418.241,432.257,44
6.273,448.251,460.289,462.267,476.283,490.299,492.277,504.315,506.293,520.3
09,534.325,548.341)
Dim dRange(2) As Double
      dRange(1) = 360.0
      dRange(2) = 549.0

Dim bit As Integer
Dim p As MassPeakInfo
Dim maxint As Double
Dim s,c As String

Open "D:\Data\mPEG_Izoc\read.txt" For Output As #2

For i = 1 To nSpectra
  aAnaSpec(i).oSpectrum.Process(faFindPeaks, dRange)
  Dim j, ref As Long
  Dim m As MassList
  Set m = aAnaSpec(i).oSpectrum.MassList
  maxint=0
  For j=1 To m.Count
    If m(j).Intensity>maxint Then maxint=m(j).Intensity
  Next j

  s=""
  For ref=0 To 19
    c="0"
    For j=1 To m.Count
        Set p = m(j)
      If                 p.MassToChargeValue>rp(ref)-0.1                 And
p.MassToChargeValue<rp(ref)+0.1 And p.Intensity>maxint/4 Then c="1"
    Next j
    s=s+c
  Next ref
  Print #2, s
Next i
Close #2

End Sub
```

Codes for encoding the text

Character	m				
	3	4	5	6	7
space	0	0	0	0	0
e	1	0	0	0	0
t	0	1	0	0	0
a	0	0	1	0	0
o	0	0	0	1	0
i	0	0	0	0	1
n	1	1	0	0	0
s	1	0	1	0	0
h	1	0	0	1	0
r	1	0	0	0	1
d	0	1	1	0	0
l	0	1	0	1	0
c	0	1	0	0	1
u	0	0	1	1	0
m	0	0	1	0	1
w	0	0	0	1	1
f	1	1	1	0	0
g	1	1	0	1	0
y	1	1	0	0	1
p	1	0	1	1	0
b	1	0	1	0	1
v	1	0	0	1	1
k	0	1	1	1	0
j	0	1	1	0	1
.	0	1	0	1	1
x	0	0	1	1	1
q	1	1	1	1	0
z	1	1	1	0	1
,	1	1	0	1	1
ó	1	0	1	1	1
capital letter	0	1	1	1	1
enter	1	1	1	1	1
